# Microvascular Invasion in Hepatocellular Carcinoma: Some Puzzling Facets

**DOI:** 10.5152/tjg.2024.22769

**Published:** 2024-02-01

**Authors:** Ibrahim Umar Garzali, Brian I. Carr, Volkan İnce, Burak Işık, Ayşe Nur Akatlı, Sezai Yılmaz

**Affiliations:** 1 Liver Transplant Institute, İnönü University Faculty of Medicine, Malatya, Turkey; 2 Department of Surgery, İnönü University Faculty of Medicine, Malatya, Turkey; 3 Department of Pathology, İnönü University Faculty of Medicine, Malatya, Turkey

**Keywords:** Hepatocellular carcinoma, microvascular invasion, survival, size

## Abstract

**Background/Aims:**

Hepatocellular carcinoma is the main type of primary liver cancer. Macroscopic vascular invasion is usually identified during imaging, whereas microvascular invasion is usually determined by histopathological evaluation. We aim to identify the association between microvascular invasion and other markers of tumor aggressiveness and to identify the role of microvascular invasion in the prognosis of patients who were treated by liver transplantation for hepatocellular carcinoma.

**Materials and Methods:**

This is a single-center retrospective analysis of prospectively collected data. Patients who received liver transplantation for hepatocellular carcinoma were included in the study. Data were collected regarding sociodemographic variables, criteria of selection for liver transplantation, pretransplant alpha-fetoprotein, presence or absence of microvascular invasion, presence or absence of recurrence, overall survival, and disease-free survival. Data were analyzed using Statistical Package for the Social Sciences.

**Results:**

Sociodemographic laboratory values and radiologic tumor characteristics were found to be similar in patients with or without microvascular invasion. Our study revealed that microvascular invasion is associated with increased recurrence, decreased diseased-free survival, and decreased overall survival, only for patients with hepatocellular carcinoma beyond Milan criteria at the time of liver transplantation.

**Conclusion:**

For patients beyond Milan criteria, but not within Milan criteria, microvascular invasion plays a significant role in predicting recurrence and shorter survival after liver transplantation.

**Main Points**

- Microvascular invasion is a poor prognostic feature in hepatocellular carcinoma.
- Microvascular invasion is associated with increased recurrence, decreased diseased-free survival, and decreased overall survival.
- For patients within Milan criteria, microvascular invasion is not associated with poor prognosis.

## Introduction

Hepatocellular carcinoma (HCC) is the main type of primary liver cancer, accounting for 90% of all primary liver cancers.^1^ It is associated with significant heterogeneity and poor prognosis which is why it is the third most common cause of cancer-related mortality worldwide.^2,3^ According to the annual forecast of the World Health Organization, more than 1 million patients will die of HCC by 2030.^4,5^

There are multiple options for the treatment of HCC but surgical resection and liver transplantation are still the most effective treatments.^6^ Recurrence is one of the important factors affecting the long-term survival of patients after surgical treatment. The 5-year recurrence rates after surgical resection and liver transplantation are as high as 70% and 35%, respectively.^7^ Some parameters are considered to predict recurrence and disease-free survival. These parameters are considered the markers of tumor aggressiveness and poor prognostic factors in HCC. They include laboratory parameters such as elevated alpha-fetoprotein (AFP) and gamma glutamyl transferase (GGT), pathologic parameters such as microscopic vascular invasion (MVI) by tumor, multiple tumor nodules and degree of tumor differentiation.^7-10^

Vascular invasion in HCC can be considered to be either macroscopic or microscopic. Macroscopic vascular invasion can usually be identified during imaging evaluation, whereas, MVI, is usually determined by histopathological evaluation of the resected specimen or explanted liver.^8^

Preoperative prediction of MVI in HCC remains elusive with some studies revealing that MVI can be predicted by some serum markers, such as des-gamma-carboxyprothrombin, AFP, or peripheral neutrophil-to-lymphocyte ratio (NLR). Other markers that may predict MVI include tumor size, multiple tumor nodules, and capsular invasion.^11-14^

The aim of this study was to identify the association between MVI and other markers of tumor aggressiveness and to identify the role of MVI in the prognosis of patients who were treated by liver transplantation for HCC.

## Materials and Methods

### Study Design and Study Location

This is a single-center retrospective analysis of prospectively collected data. The study was carried out in Liver Transplantation Institute, İnönü University, Malatya, Turkey. Consecutive patients who received liver transplantation for HCC were included in the study. Follow-up protocol for patients who underwent transplantation for HCC in the center was described in a previous publication.^10^

### Study Population

#### Inclusion criteria:

Consecutive patients that had liver transplantation for HCC

#### Exclusion criteria:

Patients with tumors of >10 cm or presence of macroscopic PVT.

#### Data Collection

Data were collected regarding age, gender, presence or absence of cirrhosis, cause of cirrhosis, number of nodules, criteria of selection for liver transplant, maximum size of the tumor, pretransplant Platelet-Lymphocyte Ratio (PLR), pretransplant neutrophil-lymphocyte ratio (NLR), pretransplant AFP, pretransplant GGT, type of transplant, presence or absence of microvascular invasion (MVI), presence or absence of tumor recurrence, overall survival (OS), and disease free survival (DFS). 

#### Ethics Committee Approval

Ethical approval was obtained from the institutional review board of Liver Transplantation Institute, İnönü University, Malatya, Turkey (Approval No: 2022/4006, Date: 25-10-2022). Written informed consent was obtained from all patients.

#### Statistical Analysis

Data were analyzed using Statistical Package for the Social Sciences version 25.0 (IBM Corp.; Armonk, NY, USA). The analysis was done separately for patients within Milan group and those beyond Milan group. Quantitative variables are expressed as median (range), mean ± SD or mean ± standard error of mean. Qualitative variables are expressed as ratio and percentage. Patients were also grouped into 2 based on presence or absence of MVI. The sociodemographic characteristics, liver enzymes, survival, recurrence and tumor characteristic of the 2 groups were compared. One-way analysis of variance with test of heterogeneity was used to compare quantitative variables between those with MVI and those without MVI with *P* < .05 considered significant.

Cutoffs for quantitative variables were identified by constructing receiver operating characteristic curve. The variable generated by grouping the quantitative variable was then compared between those with MVI and those without MVI using univariate analysis with *P* < .05 considered significant. Those with *P* < .05 were then subject to multivariate analysis. Survival analysis was conducted using the Cox regression analysis to identify independent risk factors predicting OS and DFS.

### Results

From April 2006 to May 2022, a total of 3226 liver transplants were performed at Liver Transplantation Institute, İnönü University, Malatya, Turkey. Out of these, 406 (12.6%) were transplanted for HCC. Overall, MVI by tumor was found on pathology of 138 patients (34.0%).

Of 406 patients who received liver transplantation for HCC in our institute, only 271 fulfilled the inclusion criteria of tumor less than 10 cm with absence of macrovascular invasion and they were included for analysis. Two hundred six of these patients were within Milan while 65 of them were beyond Milan. Among the patients analyzed, 89 had MVI; 44 of these patients (49.4%) were beyond Milan, while 45 patients (50.6%) were within Milan.

#### Effect of Microvascular Invasion on Survival After Liver Transplantation for Hepatocellular Carcinoma

**Figure 1.**
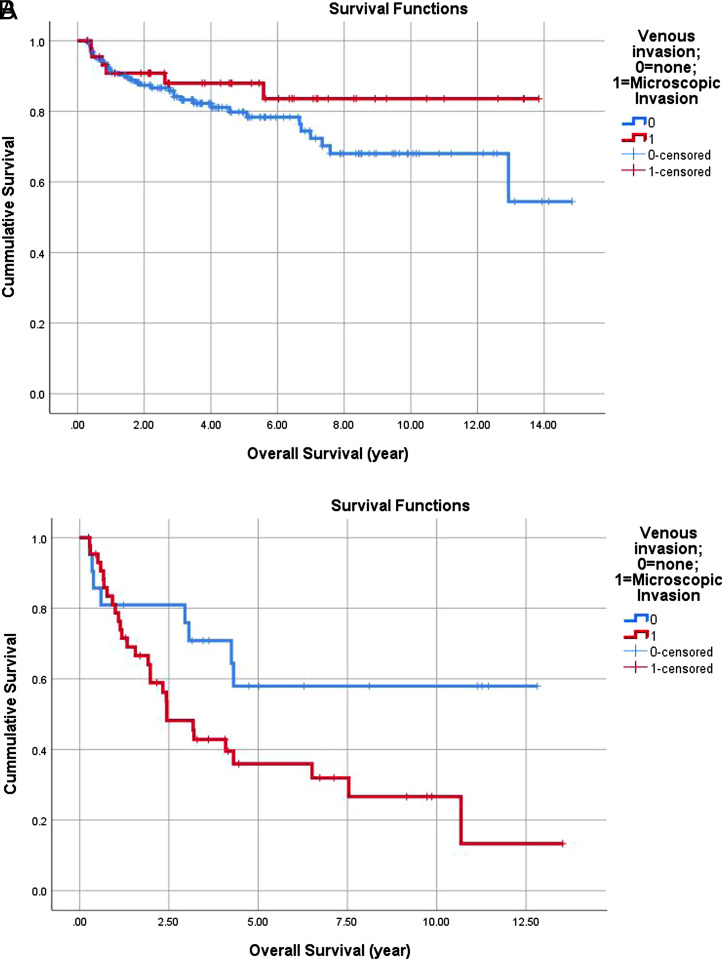
(A) Kaplan–Meir curve comparing OS in patients with and without MVI within Milan 225 × 133 mm. (B) Kaplan–Meir curve comparing OS in patients with and without MVI beyond Milan.

A survival analysis of patients within Milan criteria using Kaplan–Meier method showed no difference in OS between patients with or without MVI, log rank *P* = .196. For patients who were beyond Milan criteria at the time of liver transplantation, there was a statistically significant difference in OS between patients with and without MVI (*P* = .043, HR = 0.440) (Figure 1A and 1B).

**Figure 2.**
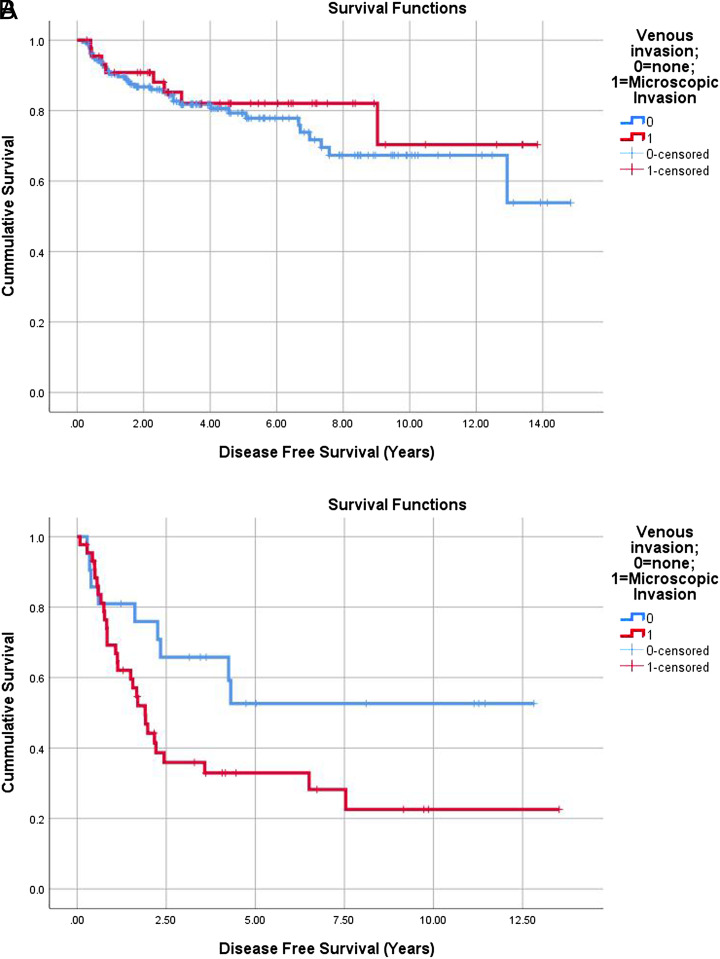
(A) Kaplan–Meir curve comparing DFS in patients with and without MVI within Milan. (B) Kaplan–Meir curve comparing DFS in patients with and without MVI beyond Milan.

We also found that for patients beyond Milan, presence of MVI at the time of transplant had a shorter disease-free survival compared to patients without MVI as shown in Figure 1 (*P* = .047, HR = 0.465) However, for patients within Milan, there was no difference in DFS between those with MVI and without MVI (*P* = .439) (Figure 2A and 2B).

#### Comparison of Tumor Characteristics Between Those with Microvascular Invasion and Those Without Microvascular Invasion

Our study revealed that for patients within the Milan criteria, there is a statistically significant difference between the tumor characteristics of those with MVI and those without MVI. Patients with MVI tend to have higher maximum tumor diameter (MTD), higher pretransplant AFP levels, higher number of tumor nodules, and higher number of patients with poorly differentiated tumors. For patients beyond Milan criteria, there was no statistically significant difference between those with MVI and those without MVI with respect to MTD, AFP, or levels of tumor differentiation. However, for patients with MVI, they tend to have multinodular tumors compared to without MVI (Table 1).

#### Microvascular Invasion and Recurrence of Hepatocellular Carcinoma After Liver Transplantation

The role of MVI in predicting recurrence after liver transplantation for HCC was assessed and we found that for patients within Milan criteria, there was no difference between patients with and without MVI in term of recurrence. There was tumor recurrence in 7 patients (3.4%). Four of these patients were without MVI while 3 had MVI. The difference was not statistically significant with *P* = .998. 

For patients beyond Milan criteria, we found that MVI is an independent predictor of recurrence and this association is shown in Table 2 (*P* = .026). We also found that patients with MVI at the time of transplant had a shorter disease-free survival compared to patients without MVI as shown in Figure 1 (*P* = .047, HR = 0.465).

We also analyzed tumor factors that may predict presence of MVI for patients beyond Milan criteria and we found that tumor of >5 cm. Multifocality, AFP >100, and level of tumor differentiation were not associated with MVI (Table 3).

#### Baseline Sociodemographic Variables Among the 2 Groups

The baseline sociodemographic characteristics were compared in both patients within Milan and those beyond Milan criteria. There was no statistically significant difference between those with MVI and those without MVI regarding age, gender, model for endstage liver disease (MELD) score and Child–Turcotte–Pugh score.

#### Baseline Liver Function Tests Among the 2 Groups

The baseline liver functions of the patients were compared in both patients within Milan and beyond Milan criteria. The was no statistically significant difference between patients with MVI and those without MVI when we compared serum levels of transaminases, alkaline phosphatase, total bilirubin, GGT, and albumin.

### Discussion

Microvascular invasion is so far a pathological diagnosis that is characterized by microscopic appearance of nests of tumor cells lining the vascular cavities of endothelial cells or portal and hepatic venous systems. It is usually confirmed by the identification of tumor cells within endothelial lined spaces on standard hematoxylin and eosin staining.^8^

The incidence of MVI in HCC ranges between 15% and 57.1%.^8,15^ Cong et al^9^ recommended that MVI should be stratified to reflect the increased risk of recurrence and shortened survival. They recommend the stratification based on the number and distribution of sites of MVI as follows: M0, no MVI; M1 (low risk), MVI <5 and at ≤1 cm from the adjacent liver tissue; and M2 (high risk), MVI >5 or at > 1 cm from the adjacent liver tissue. In our study, the incidence of MVI was 34.0% and it is within the range reported by Rodríguez-Perálvarez et al^16^ in their systematic review.

Microvascular invasion is a poor prognostic marker and a marker of aggressiveness in HCC. It is found to be associated with other markers of tumor aggressiveness. Lei et al^12^ and Carr et al^17^ reported a direct correlation between tumor diameter, multifocality, level of tumor differentiation, and level of pretransplant AFP and MVI. In the study by Lei et al^12^, they reported that MVI is observed in patients with large tumor diameter, multifocal tumor, poorly differentiated tumor, and serum AFP greater than 20 ng/mL. The role of tumor diameter and AFP in predicting MVI was also studied by Xiong et al^13^ and they found that tumors of >5 cm and AFP of 400 kU/L (484 ng/mL) were associated with MVI. This is similar to the findings of Yanhan et al.^18^ Hong et al^19^ in a meta-analysis confirmed the predictive role of larger tumor diameter of >5 cm and multimodality in predicting MVI.

The role of serum AFP in predicting MVI was further confirmed by Hu et al^11^ in their study in which they reported that AFP of >15 ng/mL is an independent predictor of MVI in patients with HCC. Multifocality and presence of satellite nodules were reported as a predictor of MVI by Granata et al.^14^ In our study, patients beyond Milan criteria showed no statistically significant difference in MTD, pretransplant AFP, and level of tumor differentiation between patients with MVI and those without MVI. However, when we assessed the number of tumor nodules, patients with MVI tend to have higher number of nodules compared to patients without MVI.

One of the major limitations to surgical management of HCC is that the 5-year recurrence rates after surgical resection and liver transplantation are as high as 70% and 35%, respectively.^7^ Microvascular invasion was considered to be an independent predictor of recurrence after resection or liver transplantation for HCC.^7,11,13,15^ The role of MVI in predicting recurrence after resection for HCC was studied by Iguchi et al^20^ who found that patients with MVI had higher recurrence rate and shorter recurrence-free survival. A meta-analysis conducted by Chen et al^15^ also confirmed that patients with MVI had a higher recurrence rate and shorter disease-free survival after surgical therapy for solitary, small HCC. Nitta et al^21^ and Rodríguez-Perálvarez et al^16^ also reported similar findings. Microvascular invasion was also reported to affect OS after resection or transplantation for HCC. This was supported by studies conducted by Nitta et al^21^, Donat et al^22^ and Vilchez et al.^23^

In our study, we found that for patients within Milan criteria, there was no difference between patients with MVI and without MVI in terms of recurrence, DFS, and OS. However, for patients beyond Milan criteria, we found that MVI is an independent predictor of recurrence. We also found that patients with MVI at the time of transplant had a shorter disease-free survival and OS compared to those without MVI. However, there have also been reports that highlighted the limited role of MVI in predicting recurrence, OS, and DFS for HCC below specific size. Chan et al^24^ reported no statistically significant difference in recurrence, DFS, and OS of patients with or without MVI if they are within Milan criteria or up to 7 criteria that received LT, similar to our results reported here. El-Fattah^25^ reported that for tumors of <2 cm, MVI does not affect survival or recurrence. This is different from the initial reports of Mazzaferro et al^26^ in 2009 where they reported that presence of MVI is associated with increased recurrence rate and decrease OS even in patients within Milan criteria. Similarly, in a recent meta-analysis, Chen et al^15^ reported that in tumors less than 5 cm, MVI is associated with worse DFS and OS. 

Reports on the prognostic role of MVI in patients beyond Milan criteria are less contradictory as most reports indicated worse DFS and OS for patients with MVI beyond Milan criteria. Thus, Pommergaard et al^27^ reported worse OS and DFS for patients with MVI that were beyond Milan criteria and up to 7 criteria. This was also supported by the study of Gundlach et al.^28^

The reason for the different prognostic roles of MVI in different groups of patients with HCC is not fully understood but it might possibly be related to other factors, such as the level of circulating tumor cells (CTCs). Circulating tumor cells are released into the circulation from the primary tumor. Previous studies have demonstrated that CTCs serve a key function in metastasis and recurrence in HCC after surgical resection and liver transplantation.^29-31^ This role of CTC in predicting recurrence after surgical treatment of HCC is associated with preoperative level of CTCs. Xue et al^30^ reported that the presence of pretransplant CTCs of greater than or equal to 5/7.5 mL of blood is associated with increased recurrence and shortened DFS (with a hazard ratio of 5.142) especially in the presence of MVI. The level of pretransplant CTCs is directly related to tumor size and tumor stage.^32^ Court et al^32^ reported that for patients within Milan or UCSF criteria, the median number of pretransplant CTCs was 3/7.5 mL of blood. For patients with tumors beyond the specified criteria, Court et al^32^ reported median CTCs of 9/7.5 and 12/7.5 mL of blood, respectively, for locally advanced and metastatic HCC, respectively. The association between CTCs and tumor size was also reported by Chen et al^33^ and in their study they also reported that level of CTCs is directly correlated with tumor size in patients with HCC. 

Our findings suggest at least 2 areas that need clarification and are not addressed in this study. First, if MVI is associated with increased recurrence and shortened survival, as we found for our beyond Milan patients, then why are there no recurrences or shortened survival in our within Milan patients who had MVI? There must be some other factor(s) that predispose to recurrence of which we were unaware and therefore could not take into account in the Milan patients who had MVI. The tumors of patients with MVI must have access to the portal circulation, by definition, and thus to the systemic circulation. Second and conversely, how do we explain the recurrences and decreased OS in patients beyond Milan with MVI? The clinical laboratory characteristics, MTD, and AFP levels were not found to be significantly different for patients with or without MVI. Thus, MVI may be a necessary but not sufficient explanation for the presence of recurrences and thus decreased OS.

There are both strengths and weaknesses of our study. Strengths include the size of the study and the stratification by tumor size. Weaknesses include the absence of molecular profile or our ability to measure CTCs.

The prognostic role of MVI in HCC after liver transplant may not be universally applicable to all patients. For patients within Milan criteria, the role of MVI in predicting outcome is limited. However, for patients beyond Milan criteria, MVI plays a significant role in predicting recurrence and shorter survival after LT.

## Figures and Tables

**Table 1.** Comparison Between Tumor Characteristics of Patients with MVI and Those Without MVI Among Patients Beyond Milan

**Beyond Milan Group**			
Variable	Vascular Invasion Group	No Vascular Invasion Group	*P*
MTD (mean) cm	6.7 ± 1.6	7.2 ± 1.4	.237
AFP (median)	28.30 (1-1782)	17.20(0.4-6388)	.327
Number of nodules (mean)	5 ± 4	2 ± 2	**.005**
Level of differentiation			
­ Well differentiated	10	8	.195
Moderately and poorly differentiated	34	13	

AFP, alpha fetoprotein; MTD, maximum tumor diameter. Bold values indicate significant difference between patients with MVI and those without MVI.

**Table 2.** Association Between MVI and Recurrence for Patients Beyond Milan Criteria

	Recurrence Present	Recurrence Absent	*P*
Venous invasion absent	3	18	**.026**
Venous invasion present	19	25	

**Table 3.** Analysis of Factors Predicting MVI in Patients Beyond Milan Criteria

Variable	*P*	Odds Ratio
MTD (5 cm vs. >5 cm)	.537	0.600
Multifocality (single nodule vs. multiple nodules)	.220	0.513
Level of differentiation (poor vs. moderate vs. well)	.536	0.627
AFP (>200 vs. ≤200)	.558	0.722

AFP, alpha fetoprotein; MTD, maximum tumor diameter.
